# Co-exposure to allergen and diesel exhaust enhance inflammatory responses in human airway submucosa

**DOI:** 10.1186/1710-1492-10-S2-A57

**Published:** 2014-12-18

**Authors:** Ali Hosseini, Tillie L Hackett, Jeremy Hirota, Kelly McNagny, Chris Carlsten

**Affiliations:** 1 Department of Medicine, University of British Columbia, Vancouver, British Columbia, V6T 1Z3, Canada; 2 University of British Columbia, James Hogg Research Centre of the Heart + Lung Institute, Vancouver, British Columbia, V6Z 1Y6, Canada; 3 University of British Columbia, Biomedical Research Centre, Vancouver, British Columbia, V6T 1Z3, Canada

## Background

Asthma is a chronic condition described by inflammation of the airways and lungs. Diesel exhaust (DE) is a major contributor to ambient particulate matter (PM) air pollution. There is rising evidence that PM acts as adjuvant on the immune responses and may lead to augmentation of allergic inflammation [1,2]. We aim to elucidate if DE increases allergen-induced inflammation and cellular immune response in the airways of atopic human subjects.

## Methods

15 volunteer participants with allergy to house dust mite allergen (Der p 1), birch or Timothy grass were recruited. In a randomized fashion, subjects inhaled DE (300µg PM_2.5_/m^3^) or filtered air for 120 minutes. One hour following the exposure, the extract of an aeroallergen to which the individual is sensitive, or placebo (sterile saline), was instilled into contralateral lung segments through bronchoscopy. Endobronchial biopsies from these same segments were then acquired 48 hours after each exposure. This was repeated 4 weeks later in each subject with the alternative inhalant. Thus, biopsies under 4 different conditions were created: filtered air + saline (FAS), DE + saline (DES), filtered air + allergen (FAA) and DE + allergen (DEA). Biopsies were processed and embedded in glycol methanlacrylate acrylic resin and serial sections were cut to 2µm and used for immunostaining with monoclonal antibodies to tryptase and eosinophil cationic protein (ECP). The percent positivity and distribution of activated mast cells (tryptase+) and eosinophils (ECP+) were quantified in the bronchial submucosa by Aperio ImageScope software.

## Results

The percent positivity for tryptase expression: FAS=0.54±0.05, DES=0.51±0.18, FAA=0.63±0.24, DEA= 0.94±0.23. The percent positivity for ECP expression: FAS=0.35±0.17, DES=0.38±0.11, FAA=0.61±0.14, DEA=0.73±0.33. Data are presented as mean ± SEM (n=6).

## Conclusions

Our preliminary data suggest that DE may enhance the inflammatory response to allergen in atopic individuals. This data is novel in the context of human lung tissue.
